# Muscular VSD Device Complications: Literature Review and Possible Implications for Echocardiographic Follow-Up

**DOI:** 10.3390/jcdd13030128

**Published:** 2026-03-10

**Authors:** Micah Tatum, Thomas Casto, Amulya Buddhavarapu, Elizabeth Lyman, Alison Gehred, Benjamin Blais, Clifford L. Cua

**Affiliations:** Heart Center, Nationwide Children’s Hospital, 700 Children’s Drive, Columbus, OH 43205, USA; micah.tatum@nationwidechildrens.org (M.T.); thomas.casto@nationwidechildrens.org (T.C.); amulya.buddhavarapu@nationwidechildrens.org (A.B.); elizabeth.lyman@nationwidechildrens.org (E.L.); alison.gehred@nationwidechildrens.org (A.G.); benjamin.blais@nationwidechildrens.org (B.B.)

**Keywords:** muscular ventricular septal defect, cardiac catheterization, ventricular septal defect

## Abstract

Long-term transthoracic echocardiogram (TTE) follow-up guidelines after muscular ventricular septal defect (mVSD) device closure are vague. The primary goal of this study was to perform a literature search to characterize the type and timing of complications that occur after mVSD device placement. The search was performed in Medline (PubMed) with English language and publication date (1983 to 2024) filters applied. Studies were included if they reported on patients who underwent mVSD device closure. Studies were excluded if they reported on other types of ventricular septal defect (VSD) device closures, were review papers, or did not report outcomes after the device procedure. A total of 139 articles met the criteria (retrospective, n = 63; prospective, n = 10; case reports, n = 66), encompassing 1668 patient cases. Age at the time of mVSD closure was 10.6 + 2.7 years. Incidence of complications was 17.9% (299/1668). Maximum follow-up was 160 months. Most complications were residual shunts (40.8%, 122/299), followed by valve dysfunction (13.7%, 41/299) and arrhythmias (13.7% 41/299). The vast majority of complications occurred ≤12 months post-device placement 98.0% (293/299). Only 1.3% (4/299) of complications occurred at >12 months (mild tricuspid regurgitation, n = 2; left bundle branch block, n = 1; atrial fibrillation, n = 1). Time until complication was not reported in 0.7% (2/299) of patients (residual shunts, n = 2). All clinically significant complications diagnosed via TTE occurred <12 months post-mVSD device procedure. The utility of repeat TTE beyond one year after mVSD device closure should be reassessed if no clinical concerns are present.

## 1. Introduction

Ventricular septal defects (VSDs) are the most common form of congenital heart defects, with a prevalence of approximately 4 per 1000 live births worldwide [1]. While in the past surgical closure of VSDs was the standard of care, transcatheter closure is becoming increasingly common and recognized as an effective treatment for various types of VSDs, especially muscular VSDs (mVSDs). Recent data have shown successful closure rates in these patients with limited complications [2].

Currently, guidelines delineating definitive short-term follow-up with transthoracic echocardiogram (TTE) exist, but long-term follow-up guidelines remain nebulous [3]. ACC/AHA/ASE/HRS/ISACHD/SCAI/SCCT/SCMR/SOPE guidelines recommend TTE prior to hospital discharge, within 30 days after VSD device closure; TTE 1 year after VSD device closure; and routine TTE 2–3 years after VSD device closure. However, these guidelines state, “Scenarios related to surveillance imaging of small muscular VSDs and those following complete repair without sequelae do not address duration of follow up. This does not imply indefinite follow up in such cases, and clinicians should base this decision on available guidelines” [4]. While there are data documenting the safety and efficacy of VSD device closure, no large review data exist on the type and timing of complications of VSD device closure or the utility for long-term follow-up TTE in this population [5].

The primary goal of this study was to perform an extensive literature search to characterize the timing of complications that occur after mVSD device placement to determine the need for long-term TTE in these patients. A secondary goal was to describe the type and frequency of associated mVSD device closure-related complications.

## 2. Materials and Methods

The Institutional Review Board waived the review for this study because it was not deemed to be research on human subjects.

Medline was searched through the PubMed platform using a mix of keywords and MeSH terms suggested by the search team. The search was initially run on 10 October 2024, and rerun on 15 October 2024, to expand the date range. Limiters for English language and publication dates from 1983 to 2024 were used for the team to review, and the full search strategy is included: ((“Heart Septal Defects, Ventricular” [Mesh] OR “ventricular septal defect*” [tiab] OR “ventricular septum defect*” [tiab] OR “ventricular heart septal defect*” [tiab] OR vsd [tiab]) AND (perimembranous [tiab] OR peri-membranous [tiab] OR perimembraneous [tiab] OR membranous [tiab] OR membraneous [tiab] OR muscular* [tiab] OR muscle* [tiab])) AND (“english” [Language]). This methodology was used to obtain the broadest search possible to prevent missing any relevant manuscripts and in accordance with PRISMA guidelines for a scoping review (PRISMA Checklist in Supplementary Materials). Results were exported to Covidence for the screening process.

Studies were included if they reported on patients with a congenital mVSD who underwent mVSD device closure and described the associated outcomes. If a study included both mVSD and perimembranous or other forms of VSDs, only the data for the mVSD device closure were included. Studies were excluded if they reported on multiple mVSDs (“Swiss cheese septum”) or if they were deemed complex closures (multiple types of VSD closure).

Study characteristics were stratified by the type of study (case reports/case series, retrospective/prospective cohort studies, randomized control trial, post hoc analysis). Median and mean values were used to summarize variables as needed. Data collected for each study included gender, sample size, type of device used, complication counts, and timing of complications. Counts were used to summarize the year of publication and the country of origin for each study. Types of complications documented included arrhythmias, malposition or embolization of the device, erosion of the device, fracture of the device, associated valve dysfunction post-device placement, device-related thrombus formation, residual shunt post-device placement, and associated complete heart block. Further complications not included above were categorized as other and then commented upon as needed. The timing of complications was separated into those occurring <1 day or within the same hospitalization, ≤1 month, ≤6 months, ≤1 year, ≤5 years, ≤10 years, and >10 years from device placement. All the categories were mutually exclusive, and complications were not counted in more than one time category.

## 3. Results

**Figure 1:**
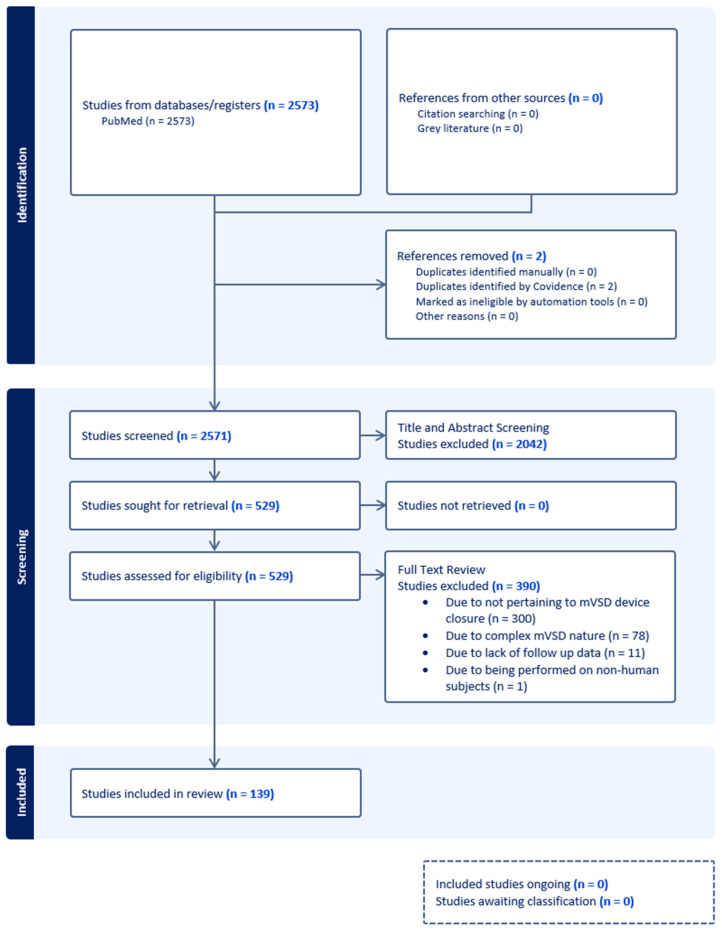
Flow chart.

Among 2573 articles retrieved and reviewed, 139 met the inclusion criteria as described above. This included 73 cohort studies (retrospective, n = 63 [2,6,7,8,9,10,11,12,13,14,15,16,17,18,19,20,21,22,23,24,25,26,27,28,29,30,31,32,33,34,35,36,37,38,39,40,41,42,43,44,45,46,47,48,49,50,51,52,53,54,55,56,57,58,59,60,61,62,63,64,65,66,67]; prospective, n = 10 [68,69,70,71,72,73,74,75,76,77]) and 66 case reports (Figure 1) [78,79,80,81,82,83,84,85,86,87,88,89,90,91,92,93,94,95,96,97,98,99,100,101,102,103,104,105,106,107,108,109,110,111,112,113,114,115,116,117,118,119,120,121,122,123,124,125,126,127,128,129,130,131,132,133,134,135,136,137,138,139,140,141,142,143]. A total of 1668 patient cases were reported in the 139 papers evaluated. The mean age at the time of mVSD device closure was 10.6 ± 2.7 years. The maximum follow-up times for the retrospective cohort studies were 160 months and 60 months for the prospective cohort studies. The maximum follow-up time for the case reports was 115 months. A wide array of VSD devices were used in the studies and included the Amplatzer VSD occlude (Abbott, Abbott Park, IL, USA), the Gore Cardioform septal occlude (Gore, Newark, DE, USA), the Shanghai Shape Memory Alloy PDA occlude (Shanghai Shape Memory Alloy Company, Shanghai, China), the Lifetech Konar-MF Occluder (LifeTech Scientific, Shenzhen, China), Nit-Occlud Le VSD Coils (PFM Medical Inc., Cologne, Germany), the Rashkind double umbrella occlude (BARD, Galway, Ireland), the Rashkind and Locke clamshell occlude (BARD, Galway, Ireland), Gianturco Coils (Cook Cardiology Inc., Bloomington, IN, USA), the Lifetech muscular VSD occlude (LifeTech Scientific, Shenzhen, China), the Occlutech muscular ventricular septal defect (Occlutech, Schaffhausen, Switzerland), the CardioSeal implant (CardioVantage, LLC, Austin, TX, USA), the MFO device (LifeTech Scientific, Shenzhen, China), Cardi-O-Fix (Stairway Medical Technology, Beijing, China), the Detachable Cook Coil (Boston Scientific, Marlborough, MA, USA), the Amplatzer Vascular Plug (Abbot, Abbot Park, IL, USA), the Hyperion VSD Muscular Occluder (Comed B.V., Heerenveen, The Netherlands/Lepu MT Company, Beijing, China), the Cocoon VSD occlude (Vascular Innovations Co., Ltd., Pleasanton, CA, USA), the P1214 PDA Occluder, the Starway Medical occlude (Stairway Medical Technology, China), the MemoPart VSD Occlusion Device (Lepu MT Company, China), and the Amplatzer PDA occlude (Abbott, Abbott Park, IL, USA).

**Figure 2:**
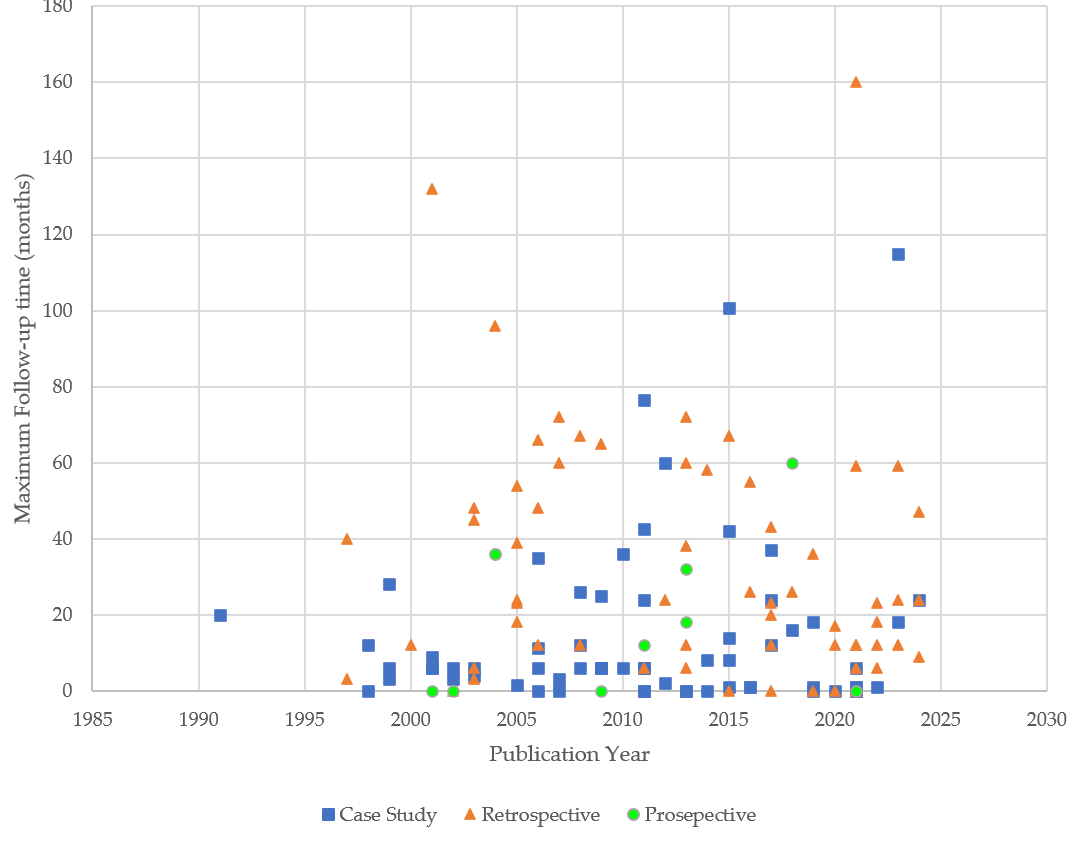
Scatter plot depicting study types across timeline of reviewed publication.

Most of the publications originated from Asia, India or the Middle East (47.5%, 66/139), with Europe (30.2% 42/139) being the next most prevalent region. The maximum range of follow-up was 0–160 months. The maximum follow-up time reported in each study logically tended to decrease the closer the study was to the present day. Study types in the early years were primarily case studies and retrospective cohort studies but came to include prospective cohort studies (Figure 2).

**Figure 3:**
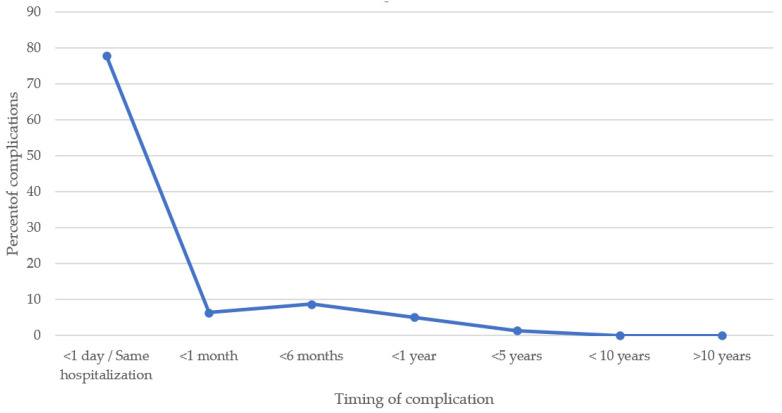
Timing of complications.

A total of 299 complications occurred in 1668 patients with an overall incidence of 17.9%. If case report complications are excluded, then the incidence of complications decreases slightly to 15.3% (248/1617). The most common types of complications were residual shunts (40.8%, 122/299) followed by valve dysfunction (13.7%, 41/299), arrhythmias (13.7% 41/299), and malposition/embolization (13.4%, 40/299) (Table 1). All 40 malposition/embolization device complications occurred during the initial hospitalization. These patients underwent device retrieval during that initial hospitalization (20 catheterization retrieval, 20 surgical retrieval). If the retrieval was via catheterization, a larger device was subsequently placed with no issues noted. If the retrieval was surgical, the mVSD were then surgically closed. Residual shunts could lead to chronic volume overload and ventricular failure as long-term complications; however, if one considers residual shunts in the immediate post-device period not as complications per se, but rather a suboptimal outcome of the procedure, the incidence of complications post-mVSD device closure could be calculated as 10.6% (177/1668). Most complications occurred <1 day or during the same hospitalization post-device placement (77.2%, 231/299). In total, 98.0% of complications (293/299) occurred ≤12 months post-device placement (Table 2, Figure 3).

Only 4/299 (1.3%) reported complications were noted at >12 months following device placement. One patient developed a left bundle branch block pattern on EKG 3 years post-device placement. Two patients were noted to have new mild tricuspid regurgitation 5 years post-device placement. This regurgitation did not worsen in either patient on follow-up studies. One patient developed atrial fibrillation and subsequently complete heart block 6 years post-device procedure. Another two (0.7%) complications occurred without documentation of timing. Both complications were residual shunts.

## 4. Discussion

When indicated and depending on anatomy and clinical circumstances, device closure of a mVSD is a reasonable option for patients [2,144]. Though short-term TTE follow-up guidelines are straightforward, guidelines for long-term surveillance with TTE are quite vague [4,145,146]. In this study, most complications occurred <1 day/within the same hospitalization post-device placement, with a preponderance of these complications occurring intra-operatively. The incidence of clinically significant structural complications >1 year post-device placement detected via TTE was 0%.

North American imaging guidelines recommend initial post-device procedure TTE, followed by a TTE at 12 months post-device placement, and then every 2–3 years post-device placement for patients with no significant complications. These guidelines did not address length of follow-up and did not imply indefinite lifetime surveillance using TTE. These decisions were left at the discretion of the primary cardiologist [4]. The 2018 ACC/AHA adult congenital heart disease VSD guidelines recommend TTE every 36 months for patients with history of a repaired VSD who are clinically doing well. However, it was unclear if these recommendations are for pre- or post-repaired VSD patients [146]. A 2023 ACC VSD follow-up algorithm recommended TTE prior to hospital discharge, 1 month post-device placement, and 6–12 months post-device placement, and then clinic visits every 2–5 years, +/−TTE every other visit or with clinical changes [3]. The 2020 European guidelines recommend clinical follow-up every 2–5 years post-device placement but make no comment on the duration of follow-up TTE [145]. No guidelines have definitively stated follow-up TTE recommendations in asymptomatic patients post-mVSD device placement.

In reviewing the available literature for mVSD device closure, >75% of the complications occurred within the initial hospitalization for device placement. This was true for all of the malposition/embolization complications, which could be considered one of the most serious complications post-mVSD device placement. This data would support guidelines recommending TTE prior to discharge so that detected complications could be addressed in a timely manner [3,4]. Complications continued to occur after initial hospitalization for device closure, but our review highlights that 98% of reported complications were noted by 1 year post-device placement. This finding supports the usefulness of the 1 year post-device TTE.

The incidence of device related complications dramatically decreased after 1 year post-device placement with a 1.3% reported incidence in this study. Of the reported complications, two were late-onset mild tricuspid valve regurgitation. For these two patients, the regurgitant jet was deemed to be hemodynamically insignificant and follow-up of these patients did not document worsening valve dysfunction. The other two complications that occurred >1 year post-device placement dealt with rhythm issues. In these two cases, TTE would not be the appropriate diagnostic test to document this complication. Two patients did not have timing of complications reported, but both complications consisted of residual shunts. It would be reasonable to assume that these residual shunts would have been noted prior to their hospital discharge. Thus, no clinically significant complication was diagnosed via TTE > 1 year post-mVSD device placement. This review raises the question of the need for TTE follow-up > 1 year post-mVSD device placement in the absence of clinical concerns.

This study had multiple limitations. The foremost limitation was the reliance on the length of follow-up for the manuscripts evaluated, given the review nature of this study. It should be noted that in manuscripts with a long follow-up period, the trend of significantly decreasing complications over time was consistent. Though a wide range of papers with the prior listed verbiage were included, only English language publications were queried; thus, there is a possibility of missing a small number of manuscripts without the included verbiage. Some studies that met the inclusion criteria may have been missed, though we also think this would be a small number considering the search criteria employed. The period under review was quite long and as such various devices listed may no longer be clinically used. Our review did not determine if certain devices were associated with complications because not all the studies were specific enough to identify these issues consistently. Detailed information about specific valves affected or arrhythmias that occurred were not uniformly reported, so no comment can be made regarding whether certain valves or arrhythmias were more common after device placement. The inclusion of case reports may have skewed the incidence of complications upward, since the numerator and denominator were essentially one. Selection and publication bias is plausible as complications have a higher likelihood of being reported. Hence, we additionally reported the incidence of complications among cohort studies to be 15%. The incidence of complications in this systematic review should be considered as at best an estimate. Finally, this study only evaluated simple mVSD device closures, so these results should not be extrapolated to other types of VSD device closures or patients with complex cardiac anatomy.

In conclusion, all clinically significant complications diagnosed via TTE occurred ≤ 12 months post-mVSD device procedure. The utility of repeat TTE beyond one year after device closure should be reassessed if no clinical concerns are present.

## Figures and Tables

**Table 1:** Types of complications.

Complications	Case Study	Retrospective	Prospective	Total Count	Percent Overall (n = 1668)	Percent Among Complications (n = 299)
Arrhythmia	3	19	19	41	2.46	13.71
Malposition or Embolization	6	29	5	40	2.40	13.38
Erosion	1	1	2	4	0.24	1.34
Fracture	0	2	0	2	0.12	0.67
Valve dysfunction	3	32	6	41	2.46	13.71
Device-related Thrombus	0	0	0	0	0.00	0.00
Residual shunt	31	76	16	122	7.31	40.80
Complete Heart Block	0	20	1	21	1.26	7.02
Other	7	18	3	28	1.68	9.36
Totals	51	197	52	299	17.93	100

**Table 2:** Timing of complications.

Complications	Case Study	Retrospective	Prospective	Total Count	Percent Overall (N = 1668)	Percent Among Complications (N = 299)
<1 day/Same hospitalization	40	147	44	233	13.97	77.93
<1 month	5	10	4	19	1.14	6.35
<6 months	3	23	0	26	1.56	8.70
<1 year	3	11	1	15	0.90	5.02
<5 years	1	3	0	4	0.24	1.34
≤10 years	0	0	0	0	0.00	0.00
>10 years	0	0	0	0	0.00	0.00
Not documented	0	2	0	2	0.12	0.67
Totals	52	196	49	299	17.93	100

## Data Availability

No new data were created.

## Supplementary Materials

The following supporting information can be downloaded at https://www.mdpi.com/article/10.3390/jcdd13030128/s1: PRISMA checklist [147].

## Abbreviations

The following abbreviations are used in this manuscript:

VSD	Ventricular septal defect
mVSD	Muscular ventricular septal defect
TTE	Transthoracic echocardiogram
